# Histone Deacetylase 6 (HDAC6) in Ciliopathies: Emerging Insights and Therapeutic Implications

**DOI:** 10.1002/advs.202412921

**Published:** 2025-04-01

**Authors:** Zhiyi Wang, Xiaofan Zhu, Zhenzhou Huang, Kaidi Ren, Jie Ran, Yang Yang

**Affiliations:** ^1^ Department of Translational Medicine Center Clinical Systems Biology Laboratories The First Affiliated Hospital of Zhengzhou University Zhengzhou 450052 China; ^2^ Center for Cell Structure and Function, Shandong Provincial Key Laboratory of Animal Resistance Biology College of Life Sciences Shandong Normal University Jinan 250014 China; ^3^ Genetics and Prenatal Diagnosis Center Department of Obstetrics and Gynecology The First Affiliated Hospital of Zhengzhou University Zhengzhou 450052 China; ^4^ Department of Pharmacy The First Affiliated Hospital of Zhengzhou University Zhengzhou 450052 China

**Keywords:** cilia, ciliopathy, HDAC6 inhibitors, histone deacetylase 6, therapeutic implications

## Abstract

HDAC6 is integral to the regulation of primary cilia, which are specialized structures that serve as crucial signaling hubs for cellular communication and environmental response. These ciliary functions are essential for maintaining cellular homeostasis and orchestrating developmental processes. Dysregulation of HDAC6 activity is implicated in ciliopathies, a group of disorders characterized by defective ciliary structure or function, resulting in widespread organ involvement and significant morbidity. This review provides a comprehensive examination of the molecular dynamics of HDAC6 in the context of ciliogenesis and ciliopathies, emphasizing its dual role in the deacetylation of microtubules and regulation of the ciliary axoneme. Furthermore, HDAC6 interacts with key signaling molecules, modulating processes ranging from cell cycle regulation to inflammatory responses, which highlights its central role in cellular physiology and pathology. The therapeutic potential of HDAC6 inhibitors has been explored, with promising results in various disease models, including retinal and renal ciliopathies, highlighting their ability to restore normal ciliary function. This analysis not only underscores the critical importance of HDAC6 in maintaining ciliary integrity but also illustrates how targeting the HDAC6‐cilia axis could provide a groundbreaking approach to treating these complex disorders. In doing so, this review sets the stage for future investigations into HDAC6‐targeted therapies, potentially transforming the clinical management of ciliopathies and significantly improving patient outcomes.

## Graphical abstract

HDAC6 regulates primary cilia, crucial for cellular signalling and environmental responses. Dysregulation of HDAC6 contributes to ciliopathies, affecting multiple organs. This review examines HDAC6's role in ciliogenesis, its interaction with signaling molecules, and its potential as a therapeutic target. HDAC6 inhibitors show promise in restoring ciliary function, offering new treatment avenues for ciliopathies.

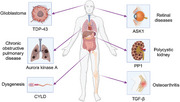

## 1 Introduction

Primary cilia are highly conserved cellular structures protruding from the cell surface and are widely found in a variety of cellular structures.^[^ ^1^ ^]^ They act as key signaling hubs, sensing mechanical and chemical signals to regulate a wide range of physiological activities.^[^ ^2^ ^]^ Abnormal cilia function can lead to a variety of serious diseases, such as retinopathy, polycystic kidney disease, obesity, and infertility, which are collectively known as ciliopathies.^[^ ^3^, ^4^ ^]^ Currently, there are various treatments for ciliopathies, including medication, gene therapy, and surgery.^[^ ^5^ ^]^ Despite the availability of various treatments, such as medication, gene therapy, and surgery, their limitations, including drug resistance, low success rates, and high recurrence rates, underscore the urgent need for more effective and reliable solutions.

More than two hundred mutations are known to cause ciliopathy,^[^ ^6^ ^]^ and the proteins encoded by these genes are involved in the maintenance of ciliary outcome and function, allowing cilia to play important roles in multiple systems of the individual. Retinal photoreceptor cells are a class of sensory neurons that convert light signals into neural responses, a process that occurs in the outer segments (OS) of the optic cone and optic rod cells. The OS is a specialized sensory cilium, and non‐syndromic ciliopathies ocular lesions are almost always associated with OS defects, such as retinitis pigmentosa and Leber congenital opsoconjunctivitis.^[^ ^7^ ^]^ Many genes, such as *usher syndrome type IIA*, encode proteins that are highly expressed not only in the retina, but also in the ciliated structures of the cochlea and vestibule so that mutations in these genes cause not only photoreceptor cell dysfunction and vision loss but also alterations in the function of cilia in the cochlea, causing auditory deficits.^[^ ^8^ ^]^ In addition to individual protein defects, ciliopathy is also caused by defects in protein complexes that are involved in cilia assembly, membrane protein sorting, and localization. For example, defects in the BBSome protein complex in *Badbid*’s syndrome (BBS) lead to ciliary abnormalities in organs such as the retina, brain, and genitalia, affecting lipid metabolism and signaling;^[^ ^9^ ^]^ *Joubert*’s syndrome (JBTS) and MORM syndrome are associated with mutations in the *inositol polyphosphate‐5‐phosphatase E* gene, which affects phosphatidylinositol metabolism and leads to abnormal ciliary function;^[^ ^10^ ^]^ and hydrocele syndrome is caused by *hydrolethalus syndrome protein 1* deletion, which affects ciliogenesis and signaling.^[^^]^ These disorders reveal that dysregulation of lipid metabolism in cilia is an important factor contributing to ciliary dysfunction and related genetic disorders.

As a more effective and advanced treatment method, targeted therapy for ciliopathies has the advantages of strong targeting ability and significant efficacy and is a promising treatment method.^[^ ^12^ ^]^ This focus on specificity makes HDAC6 an attractive target for such therapies, given its crucial role in cellular processes. HDAC6 is a crucial enzyme within the HDAC family and is characterized by its unique structure and critical role in regulating multiple cellular signaling pathways and gene expression. This enzyme is essential for various cellular functions, including cell proliferation, senescence, apoptosis, and genome stabilization, underscoring its indispensable impact throughout our lives.^[^ ^13^ ^]^

Given its critical role in fundamental cellular processes, it is not surprising that HDAC6 has been implicated in more complex diseases, particularly in the pathogenesis of ciliopathies, where recent research has shown that it plays an important role and that its overexpression can cause abnormal cell proliferation, differentiation, and apoptosis.^[^ ^14^, ^15^ ^]^ HDAC6 regulation of cilia depolymerization is influenced by a variety of proteins and pathways, such as apoptosis signal‐regulating kinase 1 (ASK1), cylindromatosis (CYLD), and Aurora A. These proteins further activate or inhibit ciliary microtubule protein deacetylation activity by phosphorylating HDAC6 or mediating HDAC6 activity by affecting its catalytic structural domain. In addition, certain proteins, such as TonEBP (Osmotic response enhancer binding protein), play important roles in this process. TonEBP acts upstream of the Aurora A – HDAC6 signaling pathway and influences matrix formation to control ciliogenesis.^[^ ^16^ ^]^ Furthermore, in certain ciliary diseases, the disintegration of cilia is associated with the upregulation and accumulation of HDAC6. The use of HDAC6 inhibitors can prevent ciliary detachment, restore ciliary function, and consequently offer a potential treatment for ciliopathies.^[^ ^17^ ^]^ Therefore, targeting the HDAC6‐cilia axis disrupts the interaction between HDAC6 and its ciliary targets, thereby preventing HDAC6‐mediated ciliary disassembly. In this review, we will explore the intricate relationship between HDAC6 and cilia and discuss the promising therapeutic potential of the HDAC6‐ciliary axis as a target for the treatment of ciliopathies.

## 2 HDAC6 Overview

Histone acetylation, a critical and dynamic regulatory process for controlling gene transcription, is governed by the interplay of various HDACs and histone acetyltransferases.^[^ ^18^, ^19^ ^]^ Specifically, HDACs play an essential role in regulating target gene expression by catalyzing the removal of acetyl groups and acetyl groups from the modified ε‐amino moiety of lysine in histone tails.^[^ ^20^ ^]^

To date, the HDAC family includes 18 members, with HDAC6 belonging to the HDAC IIb subfamily and primarily localized in the cytoplasm.^[^ ^21^, ^22^, ^23^, ^24^ ^]^ Unlike other HDACs, HDAC6 has a zinc finger motif that can interact with misfolded or polyubiquitinated proteins, causing protein degradation and aggresome formation.^[^ ^25^ ^]^ In addition, whereas most HDACs feature only one catalytic domain, HDAC6 has two domains, enabling it to not only deacetylate cytoplasmic proteins,^[^ ^26^ ^]^ such as α‐tubulin and proteins that are scarce yet significant in cancer development and progression but also engage in the biological processes necessary for cellular function, including the binding and transport of ubiquitinated proteins.^[^ ^14^, ^23^, ^27^, ^28^ ^]^ HDAC6 controls a variety of cellular processes by deacetylating and destabilizing microtubules, promoting retro translocation of ubiquitinated proteins to aggresomes, and enhancing autophagosome‐lysosome fusion,^[^ ^29^ ^]^ such as ciliary autophagy, which is essential for ciliary homeostasis.^[^ ^30^ ^]^ In terms of its functions, HDAC6 plays a vital role in anti‐neurodegenerative, anti‐cancer, and immune activities. Notably, several inhibitors have been developed and entered clinical trials.^[^ ^31^ ^]^ Extensive literature underscores that HDAC6 is crucial for various cellular functions, including cell survival, migration, structural integrity, and key signaling pathways.^[^ ^23^, ^32^ ^]^ The expression level of HDACs in normal cells is usually lower than that in tumor cells. HDAC6 is involved in key tumor cell processes, including invasion and migration, with high expression levels potentially linked to poor prognosis.^[^ ^23^, ^33^ ^]^ For example, studies have reported that histone deacetylases, including HDAC6, are involved in the development and progression of various human malignancies.^[^ ^21^, ^34^ ^]^ N^6^‐methyladenosine (m^6^A) modification plays a pro‐tumorigenic role during tumorigenesis,^[^ ^35^ ^]^ and the m^6^A methyltransferase methyltransferase Like 3 positively regulates HDAC6 translation in a m^6^A ‐dependent manner, inhibiting cellular cilia elongation and α‐microtubule protein acetylation and accelerating cancer progression.^[^ ^36^ ^]^ Therefore, HDACs, which are promising targets for cancer therapy, display potentially effective anti‐cancer properties.^[^ ^37^ ^]^

In response to other biological functions, HDAC6, as the major deacetylase of α‐tubulin, plays a key role in epithelial‐mesenchymal transition (EMT) and ciliary homeostasis,^[^ ^38^, ^39^ ^]^ and in lung fibrosis, there is no significant change in the protein level of HDAC6 but its deacetylase activity is enhanced; normally, CYLD proteins enable the HDAC6 inactivation, promoting primary ciliary homeostasis and inhibiting the EMT process; however, during lung fibrosis, CYLD defects deregulate its inhibitory interaction with HDAC6, leading to primary disassembly and EMT progression.^[^ ^40^ ^]^ Other studies have shown that cilia size and acetylated α‐microtubule protein levels are modulated during adipocyte differentiation and that HDAC6 expression is increased during adipogenesis and associated with cilia loss.^[^ ^41^ ^]^ In addition, the formyl groups of aldoses and environmental aldehydes in organisms can covalently bind to the amino or sulfhydryl groups of lipids and proteins, stimulating the inward flow of extracellular calcium and activation of the calmodulin‐Aurora A‐HDAC6 pathway, leading to the deacetylation of axonemal microtubules, which triggers cilia disassembly and contributes to the development of carbohydrate metabolism disorders.^[^ ^42^ ^]^ In conclusion, HDAC6, a crucial cellular component, plays a significant regulatory role in various cellular activities. Understanding the molecular structure and function of HDAC6 can offer insights into treating diseases associated with its dysfunction.

## 3 HDAC6 and the Cilium

### 3.1 Cilia and Ciliopathy

Cilia almost ubiquitously exist in eukaryotic cells and are hair‐like, small organelles that protrude from the surface of almost all chordate cells.^[^ ^43^, ^44^, ^45^ ^]^ The cilia can actively move, as observed in sperm, or remain stationary, as observed in photoreceptors.^[^ ^46^ ^]^ Based on whether cilia can move, they can be divided into motile and non‐motile cilia, and the non‐motile cilia are also known as primary cilia.^[^ ^46^, ^47^ ^]^ Disorders of primary ciliary function and/or construction underlie multiple human diseases and developmental disorders, such as retinal degeneration, cancer, and *Joubert* syndrome, which are collected as ciliopathies.^[^ ^44^ ^]^ The movement of cilia is impaired, resulting in primary ciliary dyskinesia, also known as motile ciliopathy, whereas diseases caused by sensory and/or signaling defects of cilia are called sensory ciliopathies. Compared with those caused by motile cilia syndrome, sensory, physiological, and developmental abnormalities caused by primary cilia defects are more diverse. For example, retinal degeneration and olfaction impairment can occur.^[^ ^46^ ^]^ In malignancies, the organelle of the primary cilium is also becoming increasingly important. Some researchers have shown that not only do primary cilia exist in benign and malignant mesothelial cells, but they can also serve as targets for the treatment of this disease.^[^ ^48^ ^]^ To understand the function of the cilium and the molecular mechanisms of ciliopathies, it is crucial to understand the composition of the primary cilium.^[^ ^49^ ^]^

It passes through dynamic transitions between assembly and disassembly to play a role in cell signaling, such as cell proliferation, tissue homeostasis, and tissue development.^[^ ^50^ ^]^ Research on the mechanism of cilia assembly and disassembly has revealed that some components, such as the small GTPase Rab7, which can serve as a regulator of cilia disassembly, have a significant effect on this process. The absence of *Rab7* can induce spontaneous ciliation in proliferating cells and promote cilia elongation during quiescence.^[^ ^50^ ^]^ In addition, the upregulation of HDAC6 caused cilia to shed. In recent years, we have made significant progress in the study of the composition and structure of cilia, but little is known about the molecular mechanisms underlying the homeostasis of cilia.^[^ ^44^ ^]^

**Figure 1:**
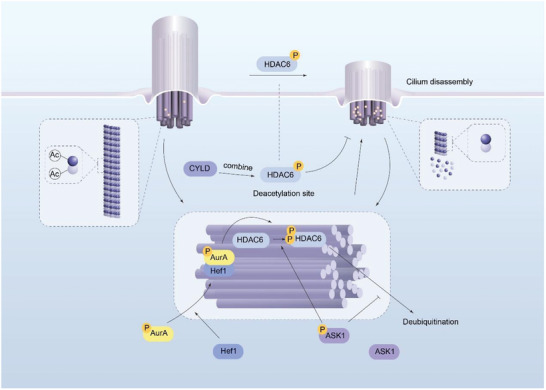
Schematic representation of HDAC6 regulation of ciliogenesis. The interaction between the HEF1 protein and ciliary matrix‐localized oncogenic Aurora A (AurA) kinase leads to the phosphorylation and activation of the microtubule protein deacetylase HDAC6, which promotes cilium disaggregation; ASK1 leads to the phosphorylation of HDAC6 at the S289 and T293 sites and inhibits HDAC6 ubiquitination; and the CYLD protein binds competitively to the HDAC6 deacetylase site, inhibiting enzyme activity and preventing cilia depolymerization.

Primary cilia assembly typically begins during the G1/G0 phase of the cell cycle, where the centrosome transforms a basal body and a ciliary axoneme.^[^ ^51^ ^]^ The assembly and extension of cilia require coordination between microtubule assembly and protein modification processes.^[^ ^51^ ^]^ Ciliary microtubules are extensively modified, with acetylation being the most common modification.^[^ ^52^ ^]^ In contrast, HDAC6, a member of the HDAC family, differs from other proteins that interact with histones in that it primarily deacetylates microtubules and contacts proteins.^[^ ^51^ ^]^ HDAC6‐mediated deacetylation of ciliary microtubule proteins results in shortening and reabsorption of cilia. Following deacetylation by HDACs, actin filaments, and contact proteins interact to promote actin filament polymerization.^[^ ^53^ ^]^ During this process, Aurora A is a mitogen‐associated kinase that does not directly affect cilium‐associated proteins but stimulates HDAC6‐dependent deacetylation of α‐microtubule proteins by phosphorylating HDAC6 and thereby activating HDAC6 at the substrate, which destabilizes the axoneme and induces rapid ciliary resorption.^[^ ^53^, ^54^, ^55^ ^]^ This mechanism involves Aurora A, which is necessary for flagellar disassembly to respond to regulated flagellar absorption signals. Aurora A activation is also dependent on multiple signaling stimuli upstream, such as the adaptor protein HEF1^[^ ^55^ ^]^ and calmodulin^[^ ^56^ ^]^ (**Figure** **1**).

Moreover, HDAC6 activity can be regulated by other mechanisms, and its interaction with other proteins can also impact its activity. For example, HDAC6 interacts with other proteins in the NOD‐like receptor family pyrin domain containing 3 inflammasome to regulate inflammasome signaling. The ubiquitin‐dependent function of HDAC6 also involves the regulation of cilia,^[^ ^53^ ^]^ and the self‐induced autophagy mechanism it mediates has been shown to induce ciliary shortening, which is referred to as “ciliary autophagy”. HDAC6 has been demonstrated to regulate primary cilial uptake under cellular stress as well as through the autophagy pathway involving autophagosome‐lysosome fusion.^[^ ^29^ ^]^ HDAC6 plays a role in a variety of biological processes, including mitosis, cancer, and heat shock, leading to cilia deletion.^[^ ^41^ ^]^ Increasing evidence suggests that HDAC6 is the main cause of ciliary detachment.^[^ ^36^ ^]^

### 3.2 Role of HDAC6 in Ciliopathy

The primary cilium is crucial for several signaling pathways, such as the Notch, Sonic Hedgehog, and platelet‐derived growth factor receptor pathways. In general, cancers, such as glioblastoma^[^ ^57^ ^]^ and lung cancer,^[^ ^58^ ^]^ can be caused by the loss of this structure. Research has shown that abnormal ciliary structure or function is also related to various pathological conditions, such as retinopathy of prematurity and cystic kidney disease.^[^ ^52^ ^]^

Interestingly, HDAC6 is a key determinant of primary cilia, regulating the length and growth of cilia, which are vital in the progression of ciliary diseases.^[^ ^52^ ^]^ Recent studies have shown that HDAC6 is localized in the ciliary base, and as an important molecule for regulating ciliary disassembly, the overexpression of HDAC6 leads to abnormalities in the cilium and membrane discs and ultimately to associated dysfunctions.^[^ ^59^ ^]^ Research by *Jie* et al. demonstrated for the first time that HDAC6‐mediated deacetylation of α‐tubulin proteins is essential for their ability to induce ciliary disassembly. In addition, if α‐tubulin or cortactin or their acetylation‐deficient mutants are overexpressed, the ability of HDAC6 to induce ciliary disassembly is increased.^[^ ^44^ ^]^ These findings indicate that reversible acetylation plays a significant role in regulating ciliary homeostasis.

Ciliopathy is a systemic whole‐organ damage disease, and the mechanisms by which aberrant expression of different proteins affects HDAC6‐regulated ciliogenesis causing different organ lesions are discussed in Figure 4. In addition to transactive response DNA binding protein of 43 kDa (TDP‐43), ASK1, Aurora, and CYLD proteins affecting HDAC6‐induced disease development described above, protein phosphatase 1 (PP1) regulates the cilium through interaction with A‐kinase anchoring protein 220 (AKAP220) and HDAC6 development and stability, which in turn affects the pathological process of polycystic kidneys. In the absence of AKAP220, PP1 is unable to anchor effectively, leading to decreased HDAC6 activity and excessive cilia development, which is associated with polycystic kidney disease development.^[^ ^60^ ^]^ In addition, primary cilia can act as signaling centers for mechanotransducer signaling pathways, so it is now believed that cilia have an important role in skeletal function, and cilia deficiency leads to osteoarthritis, tendonitis, scoliosis, and other skeletal system disorders.^[^ ^61^, ^62^ ^]^ Transforming growth factor β (TGF‐β) is a pleiotropic regulatory protein with multiple effects on osteoblast growth and differentiation.^[^ ^63^ ^]^ TGF‐β induces HDAC6 activity, which increases microtubule deacetylation in primary cilia, leading to cilia deformation and shortening as well as a decrease in the number of cells, which affects the ability of human osteoblasts to perceive mechanical stimuli, affects cell maturation, and decreases their function.^[^ ^64^ ^]^

**Figure 2:**
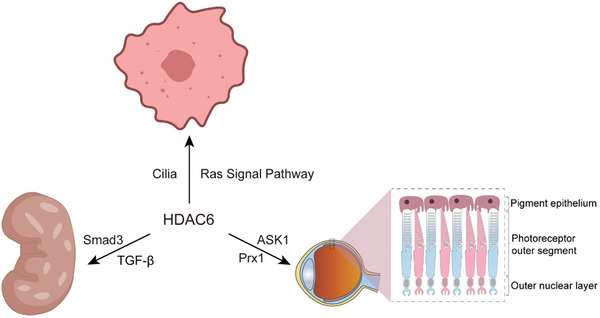
HDAC6 Effects on retina, kidney and cancer. HDAC6 Effects on retina, kidney and cancer. Abnormal regulation of HDAC6 can lead to abnormalities in the development of multiple organs, including the retina, kidney, and tumor. It affects visual function through co‐regulation with ASK1 and Prx1, promotes Smad3 signaling in renal fibrosis, and regulates tumor development by affecting the cilia and Ras signal pathways in tumors.

Dysregulation of HDAC6 often leads to abnormalities in the development of multiple organs and tissues, and most current studies have linked it to the retina, kidney, and tumors. HDAC6 is not only activated by ASK1 phosphorylation, leading to retinopathy (Figure 1)^[^ ^65^ ^]^ but also regulates the activity of peroxiredoxin 1 (Prx1), a redox‐regulated protein in cells, whose peroxide‐reducing activity is dependent on HDAC6‐mediated acetylation, affecting visual function (**Figure** **2**).^[^ ^66^ ^]^ HDAC6 also plays an important role in renal fibrosis, where TGF‐β 1 is recruited and phosphorylated when it binds to the TGF‐β receptor, and HDAC6 promotes the binding of phosphorylated Smad3 to the Smad3‐binding element, translocates to the nucleus, and drives the expression of pro‐fibrotic genes such as type I collagen.^[^ ^67^ ^]^ In tumorigenesis, HDAC6 is involved in tumorigenesis and progression by activating the Ras signaling pathway and regulating cilia length.^[^ ^36^ ^]^ Loss of cilia enhances cellular sensitivity to the Ras signaling pathway and promotes the process of tumor transformation (Figure 2).^[^ ^68^ ^]^

Researchers are currently investigating the potential of HDAC6 as a target for treating ciliary diseases, highlighting its significant molecular mechanisms in various diseases.^[^ ^53^ ^]^ Consequently, the research direction of using HDAC6 as a therapeutic target is gradually emerging as one of the most prominent topics in current research.

## 4 The Potential of Targeting the HDAC6‐Cilia Axis as a Treatment for Ciliopathy

Owing to the widespread distribution of cilia in various organ tissues, as well as their important roles in signal transmission and homeostasis maintenance, ciliopathies are complex diseases that can affect multiple organs.^[^ ^12^, ^53^ ^]^ Understanding the molecular mechanisms underlying cilia depolymerization is crucial, and HDAC6, as a key molecule in this process, mediates cilia depolymerization through mechanisms involving deacetylase and ubiquitin enzymes. This involvement positions HDAC6 as a significant player in the pathogenesis of several ciliopathies. The HDAC6‐cilium axis refers to the molecular pathway regulated by the protein HDAC6, which is vital in cilium function and ciliopathies. Research has revealed that the HDAC6‐cilium axis is regulated by different proteins in different environments.^[^ ^69^, ^70^ ^]^ Drugs targeting the HDAC6 ciliary axis have gradually been applied in the treatment of ciliopathies, including chondrosarcoma and chronic obstructive pulmonary disease.^[^ ^29^, ^71^ ^]^ Therefore, targeting this axis may provide a new strategy for the treatment of ciliopathies.

### 4.1 HDAC6‐Cilia Axis and Retinal Ciliopathy

**Figure 3:**
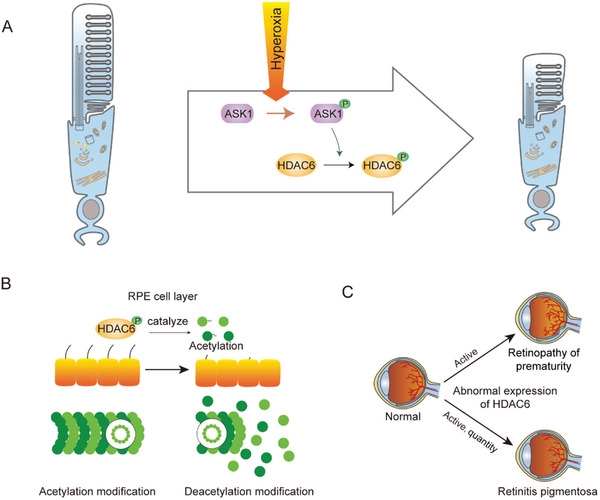
Role of HDAC6 in retinal ciliopathy. A) Description of the role of ASK1 in the regulation of HDAC6. The hyperoxic environment stimulates ASK1 and activates HDAC6, leading to cilia depolymerization and affecting retinal development. B) HDAC6 affects the supportive function of photoreceptor cells by modulating the deacetylation of cilia in retinal pigment epithelium (RPE) cells, which leads to retinitis pigmentosa. C) HDAC6 expression and activity can regulate ciliary abnormalities, leading to alterations in retinal structure and function and triggering retinopathy of prematurity and retinitis pigmentosa.

The cilium plays a crucial role in organogenesis and the function of the vertebrate eye, and its importance has been well demonstrated, particularly in photoreceptor cells.^[^ ^72^, ^73^ ^]^ Photoreceptors are polarized neurons which include rod and cone cells, and their main function is to convert light into neural signals, which are then transmitted to the brain. In photoreceptor cells, filaments are important structural components that help the cells perceive and respond to light by sensing the direction and intensity of the light.^[^ ^74^ ^]^ HDAC6 can affect the normal structure and function of photoreceptor cilia through deacetylation or ubiquitination, leading to ciliopathies of the retina. Retinal ciliopathies are caused by structural defects and/or functional impairments in photoreceptor cilia, leading to photoreceptor degeneration and vision problems. HDAC6 expression and enzyme activity are abnormal, and common ciliary diseases include retinopathy of prematurity (ROP) and retinitis pigmentosa (RP) (**Figure** **3**C).^[^ ^75^ ^]^

Premature retinal disease is caused by exposing premature babies to high‐oxygen and then normal‐oxygen environments (low‐oxygen environments), which leads to the abnormal growth of retinal blood vessels, retinal detachment, and loss of vision or visual impairment in children. The ASK1 protein is phosphorylated upon stimulation by changes in oxygen, which further phosphorylates activated HDAC6 (Figure 3A). HDAC6 induces photoreceptor cilia detachment in the early stages of ROP development, and blocking HDAC6‐mediated cilia disassembly through the intravitreal injection of small molecule compounds (tubastatin A) has a protective effect on retinal defects in ROP mice. These findings further support the pathogenic role of the HDAC6‐ciliary axis in ROP.^[^ ^59^ ^]^ In addition, targeting HDAC6 is potentially valuable in the treatment of retinopathy of prematurity, a common eye disease.^[^ ^65^ ^]^

RP is a genetic retinal disease that leads to progressive vision loss or blindness due to the degeneration or death of photoreceptor cells, and currently, no cure method can help alleviate symptoms in only patients.^[^ ^45^, ^76^ ^]^ The retina is a complex structure comprising several layers of cells. The RPE is crucial for the development and function of photoreceptors in the retina (Figure 3B).^[^ ^77^ ^]^ Research suggests that the disordered intraflagellar transport system of the cilium causes retinitis pigmentosa.^[^ ^78^ ^]^ The disassembly of RPE cilia may be an important mechanism for the occurrence and development of RP, and HDAC6 is one of the key regulatory factors of RPE cilia disassembly. Specifically, HDAC6 inhibits the formation of RPE cilia, thereby affecting normal retinal development and function. Therefore, treatment strategies targeting the HDAC6‒cilium axis may be effective approaches for treating RP.

Currently, the treatment of retinal diseases presents a dual challenge. While laser therapy and cryotherapy can effectively alleviate symptoms, they may also cause damage to retinal tissues. On the other hand, drug‐based treatments may carry potential risks, including high recurrence rates and drug toxicity. Therefore, there is an urgent need to identify a safe and effective treatment method for various ciliopathies.^[^ ^65^ ^]^

### 4.2 HDAC6‐Cilia Axis and Ciliopathies of the Kidneys

**Figure 4:**
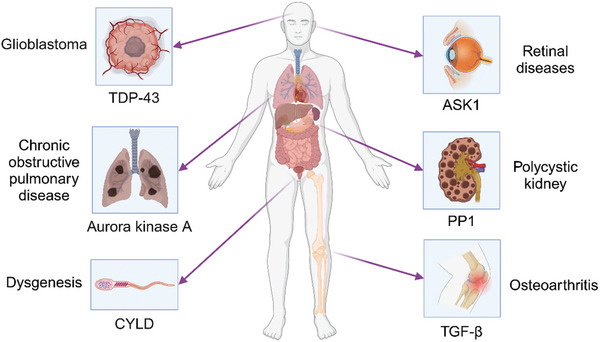
HDAC6‐cilium axis in relation to ciliopathy. Several ciliary disorders that result from abnormalities affecting the HDAC6‐cilium axis and several upstream molecules that affect HDAC6 activity are collected as ciliopathies.

Renal ciliopathies are a group of disorders associated with abnormalities in the structure or function of the primary ciliary complex and are characterized by renal tuberculosis, cystic kidney, or renal cystic dysplasia.^[^ ^79^ ^]^ Studies have shown that the expression of HDAC6 is increased in some renal diseases, such as renal fibrosis, nephrocytosis, and autosomal dominant polycystic kidney disease.^[^ ^80^ ^]^ Additionally, the expression level of HDAC6 is significantly increased in patients with nephrocytosis, and excessive expression of HDAC6 may cause abnormal proliferation of ciliated cells and abnormalities in renal structure and function.^[^ ^81^, ^82^ ^]^ Loss of tumor suppressors (e.g., VHL) has been reported in the literature to cause loss of primary cilia and initiate disease. The mitotic kinase Aurora A has novel non‐mitotic activity and interacts with the human enhancer of filamentation 1 (HEF1/NEDD9) to phosphorylate and activate HDAC6. The activation of HDAC6 leads to the disassembly of the microtubule axoneme in primary cilia, resulting in renal clear cell cancer.^[^ ^83^ ^]^ The polycystic kidney is one of the most common genetic disorders in humans, and since HDAC6 expression is upregulated in cystic epithelial cells, targeting the mechanisms affecting this change has triggered research.^[^ ^84^ ^]^ Mechanistic studies suggest that changes in HDAC6 stability and concomitant defects in actin dynamics are mediated through the A‐kinase anchoring protein AKAP220‐related protein phosphatase 1 (PP1) (**Figure** **4**).^[^ ^60^ ^]^

Additionally, HDAC6 inhibitors have shown promise in alleviating symptoms in patients with kidney ciliopathies and protecting the kidney against injury through multiple mechanisms. These findings suggest that targeting HDAC6 may provide an attractive treatment method for kidney‐related complications.^[^ ^82^ ^]^ Understanding the relationship between HDAC6 and renal ciliopathy can provide important clues for understanding the mechanism of ciliopathies of the kidneys and guiding future therapeutic research and clinical practice.

### 4.3 HDAC6‐Cilia Axis and Cancers

With the development of society, the incidence and mortality rates of cancers are also increasing annually because of factors such as environmental pollution and dietary safety. Cilia are equipped with numerous vital protein receptors and unique lipids that sense changes in the extracellular environment and transmit specific signals. Alterations in these signaling pathways may lead to decreased responsiveness to tumor therapeutic agents, which are key factors in impaired signaling in cilia‐induced malignancies.^[^ ^85^ ^]^ In human cancers, HDACs are dysregulated.^[^ ^86^ ^]^ Research on some tumors revealed that, as a cytoplasmic enzyme, HDAC6 is highly expressed in epithelial ovarian cancer, cholangiocarcinoma (CCA), glioblastoma, and other cancers. Therefore, high expression of HDAC6 is related to tumor aggressiveness.^[^ ^87^ ^]^

Histone deacetylase inhibitors are effective anticancer drugs. Vorinostat can reverse EMT;^[^ ^88^ ^]^ namely, it enhances the migration ability and antiapoptotic ability of cells. These findings identify HDACs as new targets for cancer therapy.^[^ ^58^ ^]^ However, their development is severely restricted because of certain toxic side effects. The HDAC inhibitors that have been approved by the Food and Drug Administration are all pan‐inhibitors, such as romidepsin and vorinostat, which can be used to treat cancer, but not only do they lack selectivity, they also have many side effects.^[^ ^69^, ^89^, ^90^ ^]^ However, unlike other HDAC drug inhibitors, the inhibitor of HDAC6 has been found to lack such toxicity through research, and *HDAC6*‐deficient mice also behave normally under experimental conditions.^[^ ^91^ ^]^ In addition, inhibition of HDAC6 via siRNA similarly inhibited tumor cell proliferation, migration, and colony formation.^[^ ^58^ ^]^ Therefore, the development of anti‐tumor drugs that target HDAC6 has broad application prospects.

An important link between many epithelial tumors and primary cilia loss.^[^ ^71^ ^]^ Glioblastoma multiforme (GBM) is the most aggressive and common malignant brain tumor of the neuroepithelium, and currently, the treatment for this disease involves maximal resection followed by radiotherapy and chemotherapy.^[^ ^92^ ^]^ However, despite these treatments, GBM is highly resistant to almost all therapies, and the treatments themselves can have significant side effects, leading to a low survival rate for patients.^[^ ^93^ ^]^ Therefore, there is a need to identify new therapeutic targets for GBM. For this disease, HDAC6 is a novel therapeutic target and is receiving increasing scrutiny in GBM. These findings suggest that HDAC6 and TDP‐43 have synergistic effects on GBM tumor lesions (Figure 4) and that TDP‐43 is a DNA/RNA‐binding protein that regulates neurodegenerative diseases.^[^ ^94^ ^]^ The TDP‐43‒HDAC6 axis plays a role in the stress response pathway in GBM tumorigenesis.^[^ ^95^ ^]^ In GBM cell lines, malnutrition leads to elevated expression of TDP‐43, which activates autophagy and inhibits stress‐induced apoptosis. The TDP‐43‐mediated anti‐apoptotic effect can be significantly reduced when HDAC6 inhibitors such as SAHA are used,^[^ ^95^ ^]^ which not only reveals the potential therapeutic targets of HDAC6 and TDP‐43 in GBM but also provides a scientific basis for the development of new therapeutic strategies for GBM. Moreover, HDAC6 may deacetylate and disrupt proteins required for the formation of the primary ciliary axoneme, such as acetylated α‐tubulin, thereby promoting the proliferation of GBM. HDAC6 is also overexpressed in gliomas. Blocking HDAC6 function has been shown to reduce the proliferation and promote the differentiation of ciliated glioma cells.^[^ ^96^ ^]^ Additionally, the HDAC6 inhibitors ACY‐1215 and ACY‐738 inhibited the proliferation of mouse glioma cells.^[^ ^96^ ^]^ Treatment with HDAC6 inhibitors can suppress the Hedgehog pathway (Sonic Hedgehog), restore primary cilia structure, reduce autophagy, and reduce the viability of GBM. These results suggest that HDAC6 may be a promising therapeutic target for cancer treatment.^[^ ^57^, ^58^ ^]^

Moreover, CCA is a highly aggressive tumor in which cholangiocytes typically express primary cilia that extend from the apical plasma membrane into the lumen of the bile duct. Dysregulation of multiple molecular pathways associated with the onset and progression of CCA has been linked to ciliary deletion.^[^ ^54^ ^]^ After the development of carcinoma ciliogenesis is reduced and the expression of HDAC6 molecules is detected, the use of HDAC6 inhibitors restores cilia and reduces the proliferation and invasion of bile duct cells.^[^ ^71^ ^]^ We also found that ciliary deletion in cholangiocytes disrupts multiple molecular pathways. HDAC6, a recently identified regulator, plays a crucial role in autophagy and lysosomal binding. It is involved in regulating ciliary autophagy, a major mechanism that contributes to ciliary disassembly in CCA. By inhibiting HDAC6‐mediated ciliary autophagy, CCA cell proliferation can be effectively reduced.^[^ ^17^ ^]^ Targeting HDAC6 to restore cilia has been shown to serve as a potential therapeutic approach for treating CCA.^[^ ^71^ ^]^

Primary cilia play important roles in the genesis and progression of different types of cancers, and they may function differently in different types of cancers.^[^ ^97^ ^]^ Changes in ciliary abundance, either an increase or decrease, during targeted therapy can lead to the development of resistance to treatment in cancer cells.^[^ ^98^, ^99^ ^]^

### 4.4 HDAC6‐Cilia Axis and Respiratory Ciliopathy

Initially, it was widely believed that only cilia play a role in clearing foreign objects and mucus from the respiratory tract, but with the advancement of research, it gradually became apparent that cilia have many important functions in the human body.^[^ ^78^ ^]^ Abnormalities in the structure and function of cilia in the respiratory tract lead to a reduction in their ability to clear mucus, causing ciliopathies including chronic obstructive pulmonary disease (COPD) and asthma.^[^ ^29^ ^]^ Small‐molecule inhibitors of HDAC6 are effective in treating ciliopathies such as respiratory ciliary dyskinesia, as they can reduce ciliary detachment.^[^ ^29^ ^]^

For example, in COPD, cilia dysfunction is a hallmark of this disease, and changes in cilia‐related cells and tissue processes may play a role in the pathophysiology of COPD. There is a certain relationship between COPD and HDAC6. Abnormal expression of HDAC6 may be related to the pathogenesis of COPD. Specifically, overexpression of HDAC6 may lead to proliferation and inflammatory reactions in bronchial smooth muscle cells, resulting in airway narrowing and the occurrence of COPD. In addition, HDAC6 may also affect the development of COPD by regulating the oxidative stress response and inflammatory factor expression.^[^ ^100^ ^]^ Elevated Aurora A levels produce abnormal primary cilia with ultrastructural defects. Currently, some drugs have been developed to intervene in HDAC6 and may be used to treat COPD. Some reports have shown that inhibiting HDAC6 with tubastatin A can suppress airway dysfunction caused by smoking. However, the treatment methods for this disease are not yet perfect. Therefore, HDAC6 may be a potential target for the treatment of COPD.^[^ ^29^ ^]^

**Figure 5:**
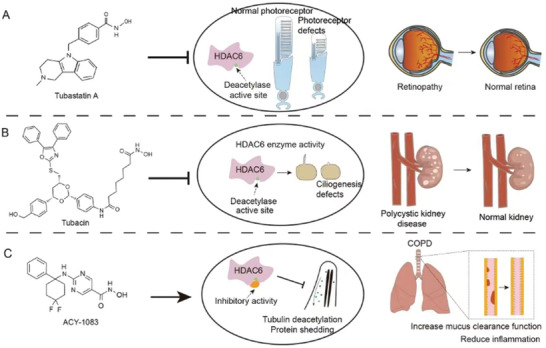
Inhibition of HDAC6 enzyme activity by the inhibitors. A) Effect of tubastatin A (an HDAC6‐specific inhibitor) on HDAC6 enzyme activity by preventing HDAC6 deacetylation and protecting the photoreceptor cilia structure. It binds tightly to the active site of HDAC6, preventing the deacetylation of its substrates, which results in a significant increase in the acetylation level of ciliary proteins, thereby enhancing microtubule stability and microtubule‐related intracellular transport processes. Pathological changes are reduced in retinopathy of prematurity and retinitis pigmentosa. B) The HDAC6 inhibitor tubacin inhibits HDAC6 enzyme activity, therefore slowing the formation of growing invasive cysts in kidneys. Tubacin is a small molecule that tightly binds to the catalytic active site of HDAC6, blocking its deacetylase activity on substrates by interacting with key amino acid residues, and selectively targets HDAC6 with minimal effects on other HDAC family members. Tubacin therefore protects renal cellular cilia structure and function and reduces cyst formation and progression in patients with polycystic kidney disease. C) ACY‐1083 is a selective, non‐hydroxamate HDAC6 inhibitor designed to tightly bind to the catalytic active site of HDAC6, blocking its deacetylase activity toward substrates. This reduces ciliary shedding, improves COPD pathological symptoms, enhances respiratory ciliary motor function, and decreases the inflammatory response.

HDAC6 has begun to be used clinically, in which Ricolinostat, an HDAC6 inhibitor, showed the potential for use in combination with nab‐paclitaxel for the treatment of patients with metastatic breast cancer in a phase Ib clinical trial, which showed that the combination therapy was safe and well‐tolerated and that the score of HDAC6 was effective in predicting the patient's response to the treatment, which offers a future precision medicine, providing a valuable biomarker.^[^ ^101^ ^]^ Another inhibitor of HDAC6, ACY‐1083, protects lung epithelial cells and attenuates TNF (tumor necrosis factor)‐induced mucin production, and may have a palliative effect on airway inflammation in COPD patients (**Figure** **5**). ACY‐1083 is stable in cell culture medium and human plasma and may have good pharmacokinetic properties. However, further validation in clinical trials is needed to determine its efficacy and safety in humans.^[^ ^102^ ^]^ Tubastatin A is a selective inhibitor of HDAC6 and may have a better safety profile than pan‐HDAC inhibitors do, as it reduces non‐specific inhibition of other HDAC isoforms, thus potentially reducing side effects. Although tubastatin A has shown low cytotoxicity in experiments, in clinical applications, prolonged and high‐dose administration may cause unknown side effects, which need to be evaluated in further clinical trials.^[^ ^103^ ^]^ Tubacin also acts as a selective inhibitor of HDAC6 and has been shown to block cyst formation in MDCK cells, an in vitro model of cystogenesis, and slow the formation of growing invasive cysts in mouse kidneys (Figure 5).^[^ ^104^ ^]^ Furthermore, tubacin does not cause G2/M cell cycle arrest, which may imply lower toxicity for clinical use.^[^ ^105^ ^]^ Additionally, A452, a selective HDAC6 inhibitor with anticancer properties, has shown promise in cancer treatment. However, its use can lead to the development of drug resistance. When combined with Dexamethasone and lenalidomide or Bortezomib, A452 has been shown to reduce cytotoxicity, enhance resistance, and improve its safety profile.^[^ ^106^ ^]^

To date, many challenges remain in the development of HDAC6 inhibitors. The typical pharmacophore model of HDACs usually consists of three structural domains: cap, linker, and zinc‐binding group,^[^ ^107^ ^]^ and most of the drug candidates suffer from poor target specificity, low drug resistance, and low brain permeability.^[^ ^108^ ^]^ Some candidate inhibitors of HDAC6 will not only target and inhibit HDAC6, but will also be highly selective for other proteins with systemic effects, e.g., ACY‐1215 also inhibits the expression of inflammatory factors, including IL‐1β and IL‐6, in human primary chondrocytes.^[^ ^109^ ^]^ In addition, ACY‐1215 inhibits inflammatory vesicle activation in hepatic injury by modulating the ATM‐actin signaling pathway;^[^ ^110^ ^]^ and ameliorates neuropathic pain and its comorbidities in peripheral nerve‐injured rats by modulating neuroinflammation.^[^ ^111^ ^]^ Thus, HDAC6 inhibitors are not limited to a single pathogenic organ.

## 5 Summary and Future Prospects

HDAC6 is a distinctive histone deacetylase that is primarily localized in the cytoplasm and is known for its ability to deacetylate a wide range of proteins and regulate various cellular functions, including protein stability, cell migration, and autophagy. Recent studies have highlighted the critical role of HDAC6 in the assembly and depolymerization of cilia, which are vital for cellular signalings and functions. HDAC6 influences cilia formation, maintenance, and function by regulating the acetylation state of α‐microtubules. This regulation is crucial for maintaining proper cellular processes and signaling pathways, such as Hedgehog and Wnt, which are essential for cell fate determination and organ development. Aberrant expression or activity of HDAC6 can disrupt these pathways, leading to the development of ciliopathies, a group of genetic disorders characterized by defective ciliary function. Clinically, the role of HDAC6 is particularly significant in diseases where ciliary dysfunction is a key factor, such as retinal ciliopathies (e.g., RP), renal ciliopathies (e.g., PKD), and respiratory ciliopathies (e.g., COPD). Under these conditions, targeting HDAC6 to modulate ciliary function has emerged as a promising therapeutic strategy. However, while the involvement of HDAC6 in cilia formation and function is well documented, the specific mechanisms by which it influences cilia‐mediated signaling in different ciliopathies remain incompletely understood. Moreover, HDAC6 is involved in many other cellular processes, raising concerns about potential non‐specific effects when targeting HDAC6 therapeutically. Therefore, developing selective HDAC6 inhibitors with minimal side effects is crucial for maximizing therapeutic efficacy in clinical settings.

Cilia themselves, as essential cellular sensory and signaling structures, play pivotal roles in various diseases when their function is impaired. In clinical practice, ciliary abnormalities have become early diagnostic markers for certain genetic disorders, cancers, and metabolic diseases. For example, in patients with polycystic kidney disease, significant changes in ciliary length and number can be detected early, offering new avenues for diagnosis and disease assessment. Additionally, the specific expression of cilia‐related proteins in certain diseases provides potential targets for novel diagnostic tools. Therapeutic strategies targeting ciliary structure and function are also gaining traction, especially in respiratory diseases such as COPD, where improving ciliary function can increase airway clearance and reduce disease severity. Cilia‐targeted therapies show significant promise in treating genetic diseases such as *Joubert* syndrome and *Bardet–Biedl* syndrome, where ciliary dysfunction is a primary pathological feature. In the context of tissue regeneration and repair, cilia are increasingly recognized for their crucial regulatory roles, particularly in organs such as the kidney, retina, and lungs. Enhancing ciliary regeneration and restoring function may be key in treating degenerative diseases. For example, in retinal diseases such as RP, promoting ciliary regeneration could be essential for preserving or restoring vision. Additionally, the influence of cilia on the immune system‐affecting immune cell migration, inflammatory responses, and tissue repair highlights their potential as therapeutic targets in autoimmune diseases, chronic inflammation, and transplant rejection. HDAC6‐targeted therapies, in particular, offer broad clinical prospects for treating ciliopathies. Inhibitors such as Tubastatin A have shown potential in repairing ciliary structures and alleviating symptoms in diseases such as ROP, RP, COPD, GBM, and CCA. However, future research should focus on developing HDAC6 inhibitors with higher selectivity and lower toxicity, ensuring their safety and efficacy over long‐term use in clinical settings. Additionally, combining HDAC6 inhibitors with other therapeutic approaches could enhance treatment outcomes, particularly in complex ciliopathies where multiple pathways may be involved.

In conclusion, both cilia‐targeted therapies and HDAC6 inhibitors represent promising avenues for treating ciliopathies. Continued research and clinical trials are essential to optimize these therapeutic strategies, ultimately improving patient outcomes and quality of life in a range of cilia‐related diseases.

## Conflict Of Interest

The authors declare no conflict of interest.

## Author Contributions

Z.W. and X.Z. contributed equally to this work. Z.W. and Y.Y. conceptualized the manuscript and created the figures. Z.W., J.R., and Y.Y. contributed to the writing of the manuscript. Z.H. and K.R. reviewed and modified the manuscript. All authors approved the final version of the manuscript.
